# A general framework for modelling the impact of co-infections on pathogen evolution

**DOI:** 10.1098/rsif.2019.0165

**Published:** 2019-06-26

**Authors:** Mary Bushman, Rustom Antia

**Affiliations:** Department of Biology, Emory University, Atlanta, GA, USA

**Keywords:** co-infection, dominance, evolutionary emergence, mathematical model, branching process

## Abstract

Theoretical models suggest that mixed-strain infections, or co-infections, are an important driver of pathogen evolution. However, the within-host dynamics of co-infections vary enormously, which complicates efforts to develop a general understanding of how co-infections affect evolution. Here, we develop a general framework which condenses the within-host dynamics of co-infections into a few key outcomes, the most important of which is the overall *R*_0_ of the co-infection. Similar to how fitness is determined by two different alleles in a heterozygote, the *R*_0_ of a co-infection is a product of the *R*_0_ values of the co-infecting strains, shaped by the interaction of those strains at the within-host level. Extending the analogy, we propose that the overall *R*_0_ reflects the *dominance* of the co-infecting strains, and that the ability of a mutant strain to invade a population is a function of its dominance in co-infections. To illustrate the utility of these concepts, we use a within-host model to show how dominance arises from the within-host dynamics of a co-infection, and then use an epidemiological model to demonstrate that dominance is a robust predictor of the ability of a mutant strain to save a maladapted wild-type strain from extinction (evolutionary emergence).

## 1. Introduction

Theoretical models of pathogen evolution frequently explore the potential for a mutant to invade a stable or growing population, or to rescue a population headed towards extinction. A key challenge in these areas is to understand how co-infection, or simultaneous occupation of a host by multiple strains or genotypes of a pathogen, influences evolutionary outcomes of interest [1]. The impact of co-infections has been most comprehensively explored with regard to pathogen virulence [2–18], but models have also considered the role of co-infections in evolution of drug resistance [19–22], vaccine escape [23] and emergence of novel human pathogens [24].

A fundamental challenge for such models is that the evolutionary consequences of co-infections depend on how co-infections operate at the within-host level, and the possibilities for the latter are seemingly endless. Co-infections may result in competitive exclusion or coexistence; virulence of co-infecting strains may compound or cancel out; factors such as cooperation, immunosuppression, immunopathology and many more come into play [2,3,14,16,17,25,26]. Consequently, existing models have not attempted to explore more than a fraction of the possibilities. Most models simply lay out a set of ‘rules’ governing co-infections, specifying key outcomes such as transmission rate, infection duration and relative transmission success of each strain. As a result, individual models tend to characterize the impact of a particular type of co-infection, or a particular aspect of co-infections, on some trait of interest. This approach can have considerable utility when modelling co-infections in a specific host–pathogen system. However, the results of one model are often not directly comparable to the results of another, due to the unique assumptions embedded in each model; consequently, we lack a general theory of how co-infections influence pathogen evolution.

Here, we argue that, for all their diversity, co-infections can be classified on the basis of one key feature which determines their epidemiological and evolutionary impact. This feature is, essentially, the fitness of a co-infection. We develop a conceptual framework which mirrors the concept of allelic dominance or heterozygous effect in genetics; under this framework, the fitness of a co-infection is shaped by the interaction between co-infecting strains, similar to how the fitness of a heterozygote depends on the interaction of two alleles. Just as the probability of fixation of a beneficial allele depends on its dominance, the probability that a mutant strain with a fitness advantage is able to invade a population depends on whether its fitness advantage is preserved in a co-infection.

At the epidemiological or between-host level, fitness is measured in terms of transmission; the accepted metric is the basic reproduction number *R*_0_, which equals the expected number of secondary infections produced by one primary infection in a susceptible host population. Suppose a host is co-infected with two strains: the wild-type and a mutant, with *R*_0_ values denoted *R*_w_ and *R*_m_, respectively. Furthermore, suppose *R*_m_ > *R*_w_. The same properties that shape the *R*_0_ of each strain (e.g. growth rate, virulence, transmissibility, etc.) determine the overall *R*_0_ of the co-infection, which we denote *R*_c_. We can express *R*_c_ as a function of *R*_w_ and *R*_m_

$$R_{c}=dR_{m}+(1-d)R_{w}.$$

The parameter *d* reflects what we call *dominance*, which captures the extent to which the higher *R*_0_ value of the mutant strain is realized in a co-infection. Note that dominance refers to how closely the co-infection fitness (*R*_0_) matches the fitness of the mutant strain; it does not reflect the ability of the mutant to ‘dominate’ at the within-host level. Thus, a mutant with *d* = 1 is fully dominant, while one with *d* = 0 is completely recessive; analogues of incomplete dominance (0 < *d* < 1), underdominance (*d* < 0) and overdominance (*d* > 1) are possible as well.

Examples of potentially dominant or recessive mutants are not difficult to imagine. For instance, a mutant could achieve a higher *R*_0_ than the wild-type via a higher growth rate that increases transmission. In a co-infection, the faster-growing mutant strain would outcompete the wild-type at the within-host level and reach densities similar to those seen in a single infection; thus, the overall *R*_0_ of the co-infection would be similar to that of the mutant by itself, making the mutant a dominant one. By contrast, consider a mutant strain that achieves a higher *R*_0_ by means of reduced virulence, resulting in a longer transmission period. In a co-infection, the virulence of the wild-type strain might cut the infection short, nullifying the higher *R*_0_ value of the mutant; that would make the mutant a recessive one. In effect, virtually any two-strain co-infection model can be mapped to a set of values for *d*, allowing scenarios of particular interest to be explored in a broader context than is possible with typical models.

This paper aims to demonstrate the utility of the conceptual framework outlined above, using evolutionary emergence as a case study of sorts. When a pathogen encounters a new and unfavourable environment, such as a newly vaccinated host population or a novel host species, its fitness may drop to unsustainably low levels, such that extinction is inevitable unless the pathogen rapidly adapts to its new environment. In a general context, this is known as evolutionary rescue [27]; in the context of infectious diseases, it is commonly called evolutionary emergence.

Here, we develop a general understanding of how co-infections influence evolutionary emergence, using the approach described above. We first use a simple within-host model to estimate dominance for several hypothetical mutant phenotypes, showing how specific co-infection models can be mapped onto a general epidemiological framework. We then use a branching process model to characterize the effects of dominance in co-infections on evolutionary emergence. We show that the likelihood of emergence increases with dominance, and that the effects of other known determinants of emergence are contingent on the dominance of the mutant strain. These results demonstrate that dominance fundamentally shapes the impact of coinfections on evolutionary emergence.

## 2. Results

### 2.1. Within-host dynamics and dominance of various mutant phenotypes

We start by showing how the dominance of a mutant strain can be calculated using a within-host co-infection model. The model, which is detailed in Methods, describes the within-host dynamics of two pathogen strains, which grow exponentially until controlled and ultimately cleared by the adaptive immune response, which behaves in a strain-transcending manner. Transmission and mortality are functions of the density of each strain. This simple two-strain model allows us to characterize five hypothetical mutant phenotypes, each of which has higher transmission potential than a baseline ‘wild-type’ strain. The extent to which this increased transmission potential is realized in a co-infection is what determines the dominance of each mutant. The hypothetical mutant phenotypes are as follows:

(1) Increased within-host replication rate $\left(r_{m}>1\right)$, resulting in a higher transmission rate.
(2) Decreased within-host replication rate $\left(r_{m}<1\right)$, resulting in decreased pathology and increased duration of transmission.
(3) Decreased virulence (v<1), resulting in decreased pathology and increased duration of transmission.
(4) Increased transmissibility, affecting only the mutant strain (*z* > 1), resulting in a higher transmission rate.
(5) Increased host infectiousness, affecting all strains in the host (x>0), resulting in a higher transmission rate.

We specify functions that govern the rate and duration of transmission based on within-host dynamics. The transmission rate is a saturating function of the total pathogen density, weighted by the parameters *z* and *x*, which relate to the efficiency of transmission. (Whereas *z* > 1 reflects a ‘selfish’ strategy that benefits only the mutant strain, x>0 indicates an ‘unselfish’ strategy that benefits both strains.) The infection lasts until the pathogen density drops below the clearance threshold, or until pathology—a linear function of pathogen density, weighted by the relative virulence of each strain—exceeds the lethal threshold. The total transmission from the infection, a quantity proportional to *R*_0_, is obtained by integrating the transmission rate over the duration of the infection.

**Figure 1.**
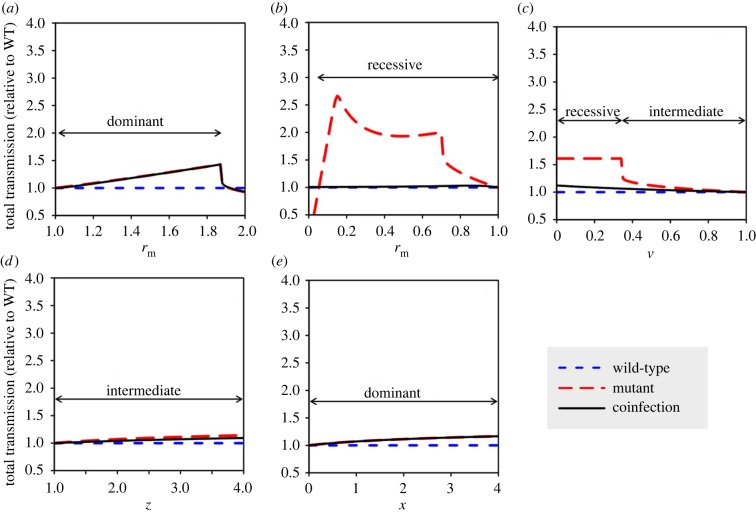
Total transmission from mutant-only single infections (red/long dash) and co-infections (black/solid), for five hypothetical mutant phenotypes. For each phenotype, transmission is normalized to the total transmission from a wild-type-only single infection (blue/short dash). (*a*) Increased within-host replication rate; (*b*) decreased within-host replication rate; (*c*) decreased virulence; (*d*) increased transmissibility; (*e*) increased host infectiousness. (Online version in colour.)

For each hypothetical mutant, we vary the parameter governing the phenotype of interest (e.g. the mutant replication rate *r*_m_) over some range, and for each value within that range, we calculate the total transmission of the mutant strain by itself as well as the total transmission from a co-infection containing both the mutant and the default wild-type strain. We then normalize these values to the total transmission of the wild-type strain by itself. Figure 1 shows the normalized total transmission of mutant-only infections and co-infections for each of the hypothetical mutant phenotypes listed above; the calculated dominance of each mutant is shown in electronic supplementary material, figure S1 and the within-host dynamics underlying non-monotonic patterns in figure 1*a–c* are depicted in electronic supplementary material, figures S2–S4. The results for each of the mutants are discussed in more detail below.

A mutant with an increased within-host replication rate (*r*_m_ > 1) attains higher densities within the host, resulting in increased transmission as long as the density does not exceed the lethal threshold (electronic supplementary material, figure S2). This type of mutant is essentially completely dominant: in a co-infection, the faster-growing mutant strain quickly overtakes the slower-growing wild-type strain, reaching densities similar to those it achieves in a single infection (electronic supplementary material, figure S2). Thus, the total transmission from a co-infection is approximately equal to the transmission of the mutant in a single infection (figure 1*a*).

By contrast, a mutant with a decreased replication rate (*r*_m_ > 1) achieves increased transmission by keeping the pathogen density below the lethal threshold, resulting in a longer duration of infection (electronic supplementary material, figure S3). This mutant is almost totally recessive because, in a co-infection, the faster-growing wild-type strain overtakes the mutant and crosses the lethal threshold, which cuts off transmission for both strains (electronic supplementary material, figure S3). As a result, the total transmission from a co-infection is roughly equal to that of the wild-type strain in a single infection (figure 1*b*).

We next consider a mutant strain with reduced virulence (*v* < 1), which allows the mutant to reach a higher density before pathology exceeds the lethal threshold. This phenotype varies in dominance, depending on whether it delays or prevents the mutant strain from crossing the lethal threshold (figure 1*c*; electronic supplementary material, figure S4). If the reduced virulence of the mutant prevents it from reaching the lethal threshold, but the lethal threshold is crossed in a co-infection with a more virulent wild-type strain, then the advantage of the mutant strain is negated, meaning the mutant is recessive. If, however, the lower virulence of the mutant simply increases the time for the lethal threshold to be reached, then this effect will be preserved to some extent when the host is co-infected with the wild-type and the mutant. The lethal threshold will be reached more quickly than when the mutant is alone, but more slowly than with the wild-type alone; thus, the overall dominance is intermediate (incomplete dominance).

Finally, we consider two mutants that increase the efficiency of transmission. The first is a mutant strain with enhanced transmissibility (z>1), which has no impact on the transmission of the wild-type; the second is a mutant that increases the overall infectivity of the host (x>0), which benefits both strains. The first mutant is almost entirely dominant; the overall transmission from a co-infection is slightly reduced since the more transmissible mutant strain suffers from competition with the wild-type (figure 1*d*). By contrast, the second mutant is completely dominant; since its effects benefit both strains equally, the total transmission from a co-infection is equal to that of the mutant alone (figure 1*e*).

So far we have focused on the total transmission from coinfections, but it is also important to know how different mutant phenotypes affect the relative transmission success of each strain. We describe these outcomes using two parameters: *c* denotes the probability of co-transmission, while *b* is the probability that, if only a single strain is transmitted, it is the mutant; thus, the overall probability of transmitting only the mutant strain is b(1−c).

**Figure 2.**
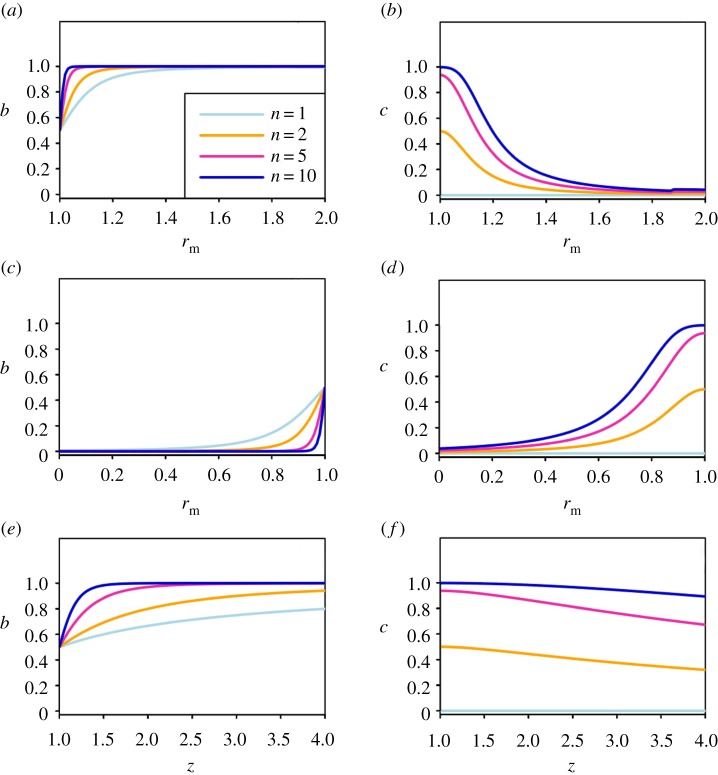
Estimates of the mutant proportion of single-strain transmission events from a co-infection (b) and the probability of co-transmission from a co-infection (c) for three mutant phenotypes. (*a,b*) Increased growth rate; (*c,d*) decreased growth rate; (*e,f*) increased transmissibility. Panels show values of *b* and *c* for transmission bottlenecks of varying size: n=1 (light blue), n=2 (orange), n=5 (pink), n=10 (violet). (Online version in colour.)

If the mutant has the same within-host fitness as the wild-type $\left(r_{m}=r_{w}\right)$ and the same transmissibility (z=1), then the strains will transmit at equal rates (b=0.5). In this case, the probability of co-transmission is simply $c=1-{\left(1/2\right)}^{n-1}$, where *n* is the size of the transmission bottleneck. In cases where the mutant strain exhibits increased transmissibility or a growth rate that is higher or lower than the wild-type, the parameters *b* (the proportion of single-strain transmission events that consist of the mutant strain) and *c* (the probability of co-transmission) are jointly determined by the bottleneck size and the within-host dynamics of the co-infection. Although there is no simple mathematical relationship between *b* and *c*, since both represent quantities integrated over the course of an infection, it will generally be the case that *c* will decrease as *b* moves further away from 0.5. Thus, larger differences between $r_{m}$ and $r_{w}$, or higher values of *z*, result in values of *b* that are closer to zero or one, which decreases the probability of co-transmission (figure 2).

### 2.2. Dominance and evolutionary emergence

We now turn to the effects of dominance on evolutionary emergence, which we explore using a multi-type branching process model. We give a brief summary of the model here; a full description can be found in Methods, with mathematical details provided in electronic supplementary material, text S1.

The model assumes that hosts can be infected with either a wild-type strain or a mutant strain, or co-infected with both strains. The *R*_0_ values of the wild-type and mutant are denoted $R_{w}$ and $R_{m}$, respectively, where $R_{w}<1$ and $R_{m}>1$. The *R*_0_ of a co-infection is denoted $R_{c}$ and is determined by the dominance (d) of the mutant strain: $R_{c}=dR_{m}+(1-d)R_{w}$. Co-infections can arise by mutation from either the wild-type or the mutant strain, with mutation probabilities $\mu_{1}$ and $\mu_{2}$, respectively. Wild-type infections leave only wild-type progeny, and mutant infections leave only mutant progeny. Co-infections can leave progeny of all three types—co-infection, mutant and wild-type—in proportions determined by the parameters *c* (co-transmission probability) and *b* (mutant proportion of single-strain transmission events).

A major advantage of branching process models is that, for a given set of starting conditions and parameters, the probability that the chain of infections goes extinct can be determined analytically. In our case, we calculate the emergence probability—which is simply the complement of the extinction probability—for chains beginning with a single wild-type infection. We then vary each of the model parameters—both individually and collectively—to explore their effects on the probability of emergence.

**Figure 3.**
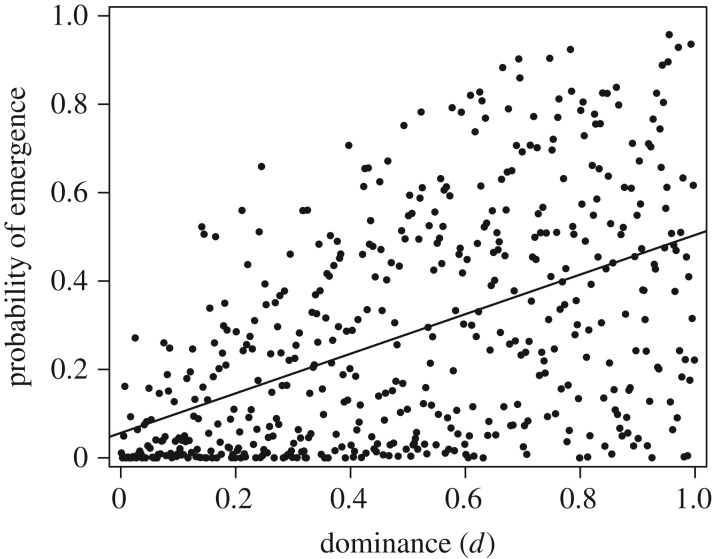
Effect of dominance (d) on the probability of emergence. Each point represents a randomly generated combination of parameters (Latin hypercube sampling, *N* = 500), with values drawn from uniform distributions on the ranges specified in table 4.

Using Latin hypercube sampling to vary all of the model parameters simultaneously, we find a strong positive correlation between dominance (*d*) and the probability of emergence (figure 3). This relationship extends to values of *d* outside the interval [0,1] (electronic supplementary material, figure S5). The explanation is intuitive: higher levels of dominance translate to higher *R*_0_ values of co-infections, which increases transmission opportunities for the mutant, resulting in a higher chance of emergence. This effect is likely to be especially strong when the mutant strain first appears, since the mutant is generated by mutation of the wild-type, resulting in a co-infection. As an outbreak progresses, the effect of dominance should diminish, unless weak transmission bottlenecks and/or high mutation rates create a preponderance of co-infections over single-strain infections.

**Figure 4.**
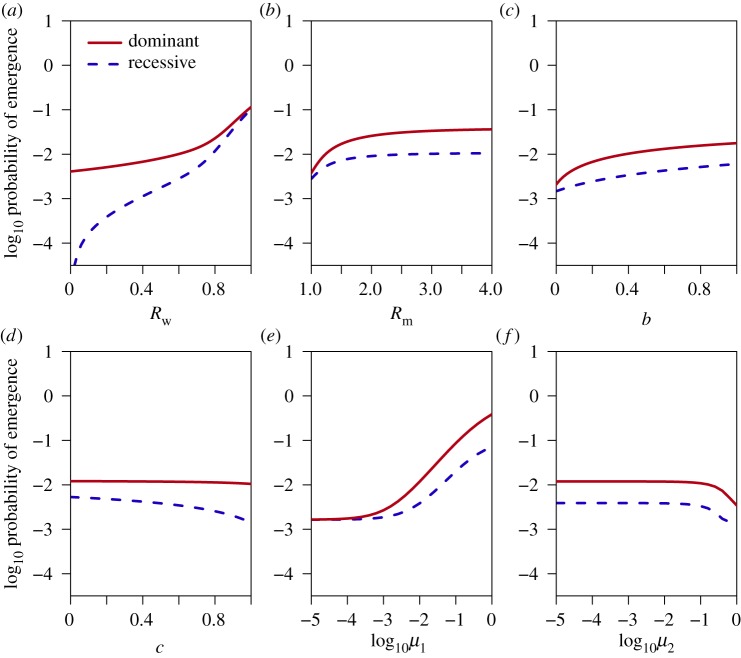
Probability of emergence as a function of individual model parameters for recessive (d=0, blue/dashed line) and dominant (d=1, red/solid line) mutants. (*a*) *R*_0_ of wild-type strain; (*b*) *R*_0_ of mutant strain; (*c*) mutant proportion of single-strain transmission from co-infections; (*d*) probability of co-transmission from co-infections; (*e*) mutation rate (wild-type to mutant); (*f*) reversion rate (mutant to wild-type). When not being varied, parameters were fixed at $R_{w}=0.75$, $R_{m}=1.5$, b=0.5, c=0.5, $\mu_{1}=0.01$ and $\mu_{2}=0.01$. (Online version in colour.)

**Figure 5.**
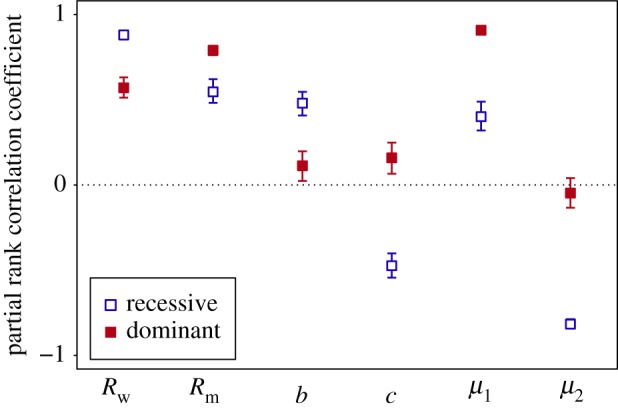
Effect of model parameters on probability of emergence for recessive (d=0, blue/open symbols) and dominant (d=1, red/filled symbols) mutants. Coefficients greater than zero indicate positive effects on emergence, while coefficients less than zero indicate negative effects. PRCCs were computed from Latin hypercube sampling (*N* = 500), with *d* fixed and other parameters varied in the ranges specified in table 4. Error bars show 95% confidence intervals based on 500 bootstrap replicates. (Online version in colour.)

Next, we consider how the other model parameters influence emergence, and how these effects depend on the dominance of the mutant strain. We explore the effects of six parameters on emergence probability: the *R*_0_ values of the wild-type and mutant strains, the probability of co-transmission, the mutant proportion of single-strain transmission events and the mutation probabilities going from wild-type to mutant and vice versa. We are interested in how these parameters interact with dominance; in the main text, we focus on comparisons between recessive and dominant mutations, while a more comprehensive analysis spanning a wider range of dominance values can be found in electronic supplementary material, figures S6 and S7. Figure 4 shows the probability of emergence as a function of each individual parameter, with other parameters held constant, while figure 5 shows the correlation between each parameter and the probability of emergence when all parameters are varied simultaneously using Latin hypercube sampling. In both figures, results are shown for recessive (d=0) and dominant (d=1) mutants.

Previous studies have shown that the probability of evolutionary rescue, or emergence, increases with the mean fitness, equivalent to the initial *R*_0_ value [28–30]. In general, the probability of extinction decreases as the mean fitness increases. It is therefore unsurprising to find that the probability of emergence increases with the *R*_0_ values of both the wild-type and the mutant (figure 4*a,b*). However, the *R*_0_ of the wild-type $\left(R_{w}\right)$ has a stronger effect on recessive mutants, while the *R*_0_ of the mutant $\left(R_{m}\right)$ has a stronger effect on dominant mutants (figure 5). This occurs because co-infections have $R_{0}=R_{w}$ if the mutant is recessive but $R_{0}=R_{m}$ if the mutant is dominant; thus, the amount of transmission from co-infections is determined by $R_{w}$ for recessive mutants but by $R_{m}$ for dominant mutants.

It has also been demonstrated that the probability of emergence increases when the mutant strain has a fitness advantage at the within-host level [31]. The closest equivalent here is the parameter *b*, which denotes the probability that, if only one strain is transmitted, that strain is the mutant. The value of *b* reflects the within-host fitness as well as transmissibility of the mutant, and indeed, we find that the probability of emergence increases with *b* (figure 4*c*). However, the effect of *b* is stronger for recessive mutants (figure 5), because a recessive mutant can only realize its higher *R*_0_ value in single-strain infections; thus, the opportunity for a mutant to escape co-infection and initiate a mutant-only infection is critical to emergence when the mutant is recessive.

Interestingly, the co-transmission probability *c* has different effects on dominant and recessive mutants (figures 4*d* and 5). Increasing *c* slightly increases the probability of emergence for a dominant mutant because it increases the proportion of secondary infections that receive the mutant strain. However, the opposite is true for recessive mutants: the probability of emergence decreases with *c* because a recessive mutant only realizes its higher *R*_0_ in the absence of the wild-type; reducing co-transmission makes this more likely by separating the mutant from the wild-type.

Previous studies have shown that higher mutation rates increase the probability of emergence by improving the chances of generating the mutant strain before the wild-type goes extinct [29,32]. Similarly, we find that the probability of emergence increases with the wild-type-to-mutant mutation probability $\mu_{1}$ (figure 4*e*). However, the effect is stronger for dominant mutants (figure 5), reflecting the fact that recessive mutants face much greater post-mutation barriers than dominant mutants. Once generated, a dominant mutant has a high chance of emergence, so mutation is effectively limiting. A recessive mutant, on the other hand, faces additional hurdles, such as the need to escape from co-infection in order for its higher *R*_0_ to be realized.

The mutant-to-wild-type reversion probability $\mu_{2}$ has no discernible effect unless $\mu_{2}$ is relatively high, in which case there is a strong negative effect on emergence for recessive mutants, but the effect on dominant mutants is much weaker (figures 4*f* and 5). The reason is that reversion (a mutant-only infection turning into a co-infection) decreases the *R*_0_ of the infection for a recessive mutant from $R_{m}$ to $R_{w}$, sharply reducing onward transmission; by contrast, reversion does not change the *R*_0_ of an infection with a dominant mutant. Only when $\mu_{2}$ is very high, is there a slight negative effect on dominant mutants (figure 4*f*), which results from the fact that the wild-type takes up a fraction of the transmission from a co-infection, which reduces onward transmission of the mutant.

**Figure 6.**
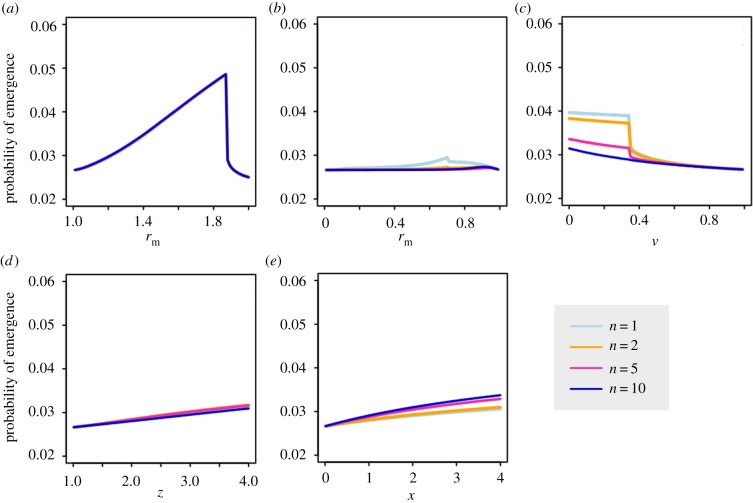
Calculated emergence probabilities for five hypothetical mutant phenotypes. (*a*) Increased within-host replication rate; (*b*) decreased within-host replication rate; (*c*) decreased virulence; (*d*) increased transmissibility; (*e*) increased host infectiousness. The output of the within-host model was used to determine values for *b*, *c* and *d*, as well as the ratio of $R_{m}$ to $R_{w}$. Values of *b* and *c* were calculated for transmission bottlenecks of varying size: n=1 (light blue), n=2 (orange), n=5 (pink), n=10 (violet). Other model parameters were fixed as follows: $R_{w}=0.9$; $\mu_{1}=0.01$; $\mu_{2}=0.01$. (Online version in colour.)

It is worth noting that, although we vary the model parameters independently to assess their effects on emergence, these parameters are not independent in reality. In particular, the mutant proportion of single-strain transmission from co-infections (*b*) and the probability of co-transmission (*c*) are related to dominance in that all three are shaped by the within-host dynamics of co-infections. To show how these parameters combine to shape emergence, we use the branching process model to calculate the probabilities of emergence for the five hypothetical mutant phenotypes discussed above (figure 6), using the output of the within-host model to provide values for *b*, *c* and *d*, as well as the ratio of $R_{m}$ to $R_{w}$. The highest probability of emergence is achieved by a mutant strain with an increased replication rate—specifically, one with a growth rate that keeps its density just below the lethal threshold (figure 6*a*). This, of course, is a dominant mutant phenotype; however, the phenotype with the next-highest probability of emergence is the one with a marked reduction in virulence (figure 6*c*), which is a recessive phenotype. Furthermore, as discussed above, this recessive mutant is highly sensitive to transmission bottlenecks, exhibiting a considerably higher probability of emergence with a strong transmission bottleneck (n=1) compared to a weaker transmission bottleneck (n=10) (figure 6*c*).

## 3. Discussion

In this paper, we construct a novel theoretical framework to explore the effects of co-infections on pathogen evolution. We argue that the critical aspect of a co-infection is its fitness (*R*_0_ value), which is a function of the fitness (*R*_0_ values) of the co-infecting strains. Drawing on concepts from genetics, we suggest that strains can be classified according to their *dominance*—i.e. the degree to which their *R*_0_ values are reflected in the *R*_0_ values of co-infections.

Using a simple within-host model, with associated functions for pathology and transmission, we show that mutant strains with various phenotypes, all of which serve to increase *R*_0_, exhibit varying levels of dominance. It is interesting to note that, among the hypothetical mutants considered here, dominance is associated with ‘gain of function’ traits—i.e. new or enhanced abilities that facilitate transmission—whereas ‘loss of function’ traits, such as reduced virulence, are found to be recessive. Intuitively, this makes sense: if the loss of some function, such as virulence, increases transmission for a mutant, then the presence of a wild-type strain that still possesses that function is likely to negate the advantage. The benefit of a new or enhanced function, on the other hand, may be diluted in a co-infection with the wild-type, but will not be completely lost unless some mechanism acts to cancel out the enhanced function of the mutant strain.

We then provide an example of how dominance in co-infections may shape evolution. We use a branching process model to explore the impact of dominance on evolutionary emergence, in which a mutant strain with *R*_0_ > 1 emerges from and ‘rescues’ a wild-type with *R*_0_ < 1 from extinction. We show that the probability of emergence exhibits a strong positive relationship with dominance, suggesting that—all else being equal—a dominant mutant is more likely to achieve emergence than a recessive mutation. However, in addition to dominance, we show that several other factors also influence the probability of emergence: the *R*_0_ values of the wild-type and mutant strains, the probability of co-transmission and the mutant proportion of single-strain transmission from co-infections and the mutation rates from wild-type to mutant and vice versa. We find that the effects of these parameters depend on dominance, with emergence of dominant mutants more sensitive to the wild-type-to-mutant mutation probability and the *R*_0_ of the mutant strain, and recessive mutants more affected by the *R*_0_ of the wild-type, the mutant proportion of single-strain transmission from co-infections, the probability of co-transmission and the mutant-to-wild-type reversion probability.

We also note that these other parameters are not independent of dominance or of one another; all are related in some way to the phenotypes of the wild-type and mutant strains. Dominance, the probability of co-transmission and the mutant proportion of single-strain transmission from co-infections are jointly determined by the within-host dynamics of co-infections. The within-host dynamics of the wild-type and mutant strains determine their *R*_0_ values, and it is likely that mutation and reversion probabilities vary depending on how many ways there are to acquire or lose a given phenotype. Thus, although dominance tends to increase the probability of emergence, the most dominant mutant phenotype does not necessarily have the highest probability of emergence. Indeed, by using the within-host model to estimate key parameters and then plugging these values into the branching process model, we show that a recessive mutant with reduced virulence may have a higher probability of emergence than other mutants with higher levels of dominance.

The results presented here show how the dynamics of co-infections can be used to identify the types of mutants that pose the greatest risk of evolutionary emergence. We show that dominance increases the probability of emergence, although this effect can be mitigated by other factors, such as the fitness of the mutant strain, the size of transmission bottlenecks and the within-host fitness and transmissibility of the wild-type and mutant strains. Furthermore, we use a within-host model to show that dominance and other key parameters arise from the within-host dynamics of co-infections, allowing the probability of emergence to be determined for specific mutant phenotypes. Based on these results alone, it is not possible to draw general conclusions about the phenotypes that are most likely to lead to emergence, because the results are likely to depend on the underlying within-host model. However, in systems where the drivers of within-host dynamics are reasonably well understood, or where empirical data on transmission of individual strains from co-infections exist, it should be possible to make robust predictions about the mutations that are most likely result in successful emergence.

Our hope is that others find this conceptual framework to be a useful way to analyse the impact of co-infection on pathogen evolution in a variety of contexts. We have shown that dominance is a robust predictor of evolutionary emergence when the wild-type is on the verge of extinction, being present in a single individual; we expect this result to generalize to situations where the wild-type is more abundant but still headed towards extinction. This would include cases where the *R*_0_ of an endemic pathogen drops below 1 due to acquired immunity, vaccination, antimicrobial drugs or other control measures. It would also apply to weakly transmissible live vaccines, such as the oral polio vaccine, which have *R*_0_ < 1 by design but can evolve higher levels of transmissibility [33–35]. Dominance may also have interesting consequences for the evolution of pathogens in stable or growing populations. For instance, a dominant mutant might be more likely to invade a population, but may also be more susceptible to invasion by cheaters that benefit from its ‘unselfish’ nature [36,37]; this may result in stable coexistence of multiple strains with different *R*_0_ values in a single population.

## 4. Methods

### 4.1. Within-host model

We use a simple within-host model to simulate co-infections with several different mutant phenotypes. The model governs the dynamics of two strains, the wild-type (W) and the mutant (M), as well as the immune response (I), through a set of ordinary differential equations, as shown below; the variables and parameters for the model are listed in tables 1 and 2

$$\frac{dW}{dt}=r_{w}W-kWI,$$

$$\frac{dM}{dt}=r_{m}M-kMI$$

$$\mathrm{and}\;\;\;\;\frac{dI}{dt}=sI\left(\frac{W+M}{\;A+W+M\;}\right).\;\;$$

**Table 1.** Variables of the within-host model: definitions.

variable	definition
W(t)	wild-type density
M(t)	mutant density
I(t)	immune response
α(t)	pathology
λ(t)	transmission
θ(t)	probability a single transmitted particle is mutant
τ	duration of infection
Λ	total transmission

**Table 2.** Parameters of the within-host model: definitions and default values.

parameter	definition	default value
$r_{w}$	wild-type replication rate	1
$r_{m}$	mutant replication rate	1
k	immune killing rate	10^−4^
s	growth rate of immune response	0.8
A	tuning parameter (sensitivity of immune response)	10^3^
x	effect of mutant on host infectivity	0
z	relative transmissibility of mutant	1
v	relative virulence of mutant	1
Φ	pathology threshold (transmission ceases when W+vM>Φ)	10^8^
Ω	clearance threshold (transmission ceases when W+M<Ω)	1
n	transmission bottleneck size	1

The densities of the wild-type and mutant strains control three key outcomes: overall (total) transmission, pathology and the relative transmission of each strain. The transmission rate is a saturating function of the wild-type and mutant densities

$$\lambda (t)=\mathrm{lo}g_{10}[\left(1+x\delta_{M})(W+zM(t)\right)].$$

The parameter *x* is the effect of the mutant on the host's overall infectivity; if x>0, then the mutant makes the host more infectious by enhancing transmission mechanisms, such as coughing or diarrhoea. This effect is dependent on the presence of the mutant; thus

$$\delta_{M}=\{\begin{array}{c} 0\;\;\;\;\;\;\mathrm{if}\;\;M=0\\ 1\;\;\;\mathrm{otherwise} \end{array}$$

Whereas *x* affects the transmission of both strains, the parameter *z* determines the transmissibility of the mutant relative to the wild-type. If z>1, then the mutant has a transmission advantage, perhaps stemming from a change in tissue tropism, that has no effect on transmission of the wild-type.

Pathology, like transmission, is a function of the densities of the two strains

α(t)=W+vM.

The parameter *v* denotes the relative virulence of the mutant relative to the wild-type, with v<1 denoting a lower level of virulence. The infection ends when pathology exceeds a set threshold (Φ) or when the combined pathogen density drops below the clearance threshold (Ω). The duration of infection is denoted τ. The total transmission, Λ, is obtained by integrating the transmission rate, λ(t), over the course of the infection

$$\Lambda =\int_{0}^{\tau }\lambda (t)dt.$$

We use $\Lambda_{w}$, $\Lambda_{M}$ and $\Lambda_{C}$ to denote the total transmission from wild-type-only infections, mutant-only infections and co-infections, respectively.

The relative transmission of each strain over the course of the infection is captured by θ(t), which is the probability that any single transmitted particle belongs to the wild-type strain

$$\theta (t)=\frac{zM(t)}{W(t)+zM(t)}.$$

With a transmission bottleneck of *n* particles, the number of mutant particles transmitted follows a binomial distribution with size *n* and probability θ(t). We are interested in the proportion of transmission events containing both wild-type and mutant particles (co-transmission), which we denote *c*, as well as the proportion of single-strain transmission events consisting of the mutant strain, which we call b

$$c=\frac{1}{\Lambda_{C}}\int_{0}^{\tau }(1-\theta {\left(t\right)}^{n}-{\left(1-\theta (t)\right)}^{n})\lambda (t)dt$$

and

$$b=\frac{1}{(1-c)\Lambda_{C}}\int_{0}^{\tau }\theta {\left(t\right)}^{n}\lambda (t)dt.$$

We use this model to characterize the effects of five hypothetical mutants on transmission in co-infections; the mutant phenotypes are listed in table 3. For each mutant, we quantify the total transmission from a wild-type-only single infection $\left(\Lambda_{w}\right)$, mutant-only single infection $(\Lambda_{M})\;$ and from a mutant-wild-type co-infection $\left(\Lambda_{C}\right)$, which we use to calculate dominance, as follows:

$$d=\frac{\Lambda_{c}-\Lambda_{w}}{\Lambda_{m}-\Lambda_{w}}.$$

**Table 3.** Hypothetical mutant phenotypes: descriptions and specific parameter values. Model parameters were set to default values listed in table 2 unless otherwise specified.

mutant phenotype	parameter values
increased growth rate	$1\leq r_{m}\leq 2,\;\Phi =10^{12}$
decreased growth rate	$0\leq r_{m}\leq 1,\;\Phi =10^{6}$
decreased virulence	$0\leq v\leq 1,\;\Phi =10^{7}$
increased transmissibility	$1\leq z\leq 4,\;\;\;\;\Phi =10^{8}$
increased host infectivity	$0\leq x\leq 4,\;\Phi =10^{8}$

**Table 4.** Parameters of the branching process model: definitions and ranges.

parameter	definition	range
$R_{w}$	$R_{0}$ of wild-type infection	$R_{w}\leq 1$
$R_{m}$	$R_{0}$ of mutant infection	$1\leq R_{m}\leq 4$
$R_{c}$	$R_{0}$ of co-infection; $R_{c}=dR_{m}+(1-d)R_{w}$	$R_{c}\geq 0$
d	dominance	0≤d≤1 (main text) −0.5 ≤ *d* ≤ 1.5 (electronic supplementary material)
c	probability of co-transmission of wild-type and mutant from a co-infection (resulting in another co-infection)	0≤c≤1
b	probability of transmitting mutant, given only one type is transmitted	0≤b≤1
$\mu_{1}$	mutation probability (wild-type to mutant)	$0\leq \mu_{1}\leq 1$
$\mu_{2}$	reversion probability (mutant to wild-type)	$0\leq \mu_{2}\leq 1$

For the mutants that have lower or higher transmission probability than the wild-type (those with changes to parameters *r*_m_ and z), we also calculate the frequency of co-transmission (c) and the proportion of single-strain transmission events that consist of the mutant strain (b) for varying values of *n* (the transmission bottleneck size).

### 4.2. Between-host model

**Figure 7.**
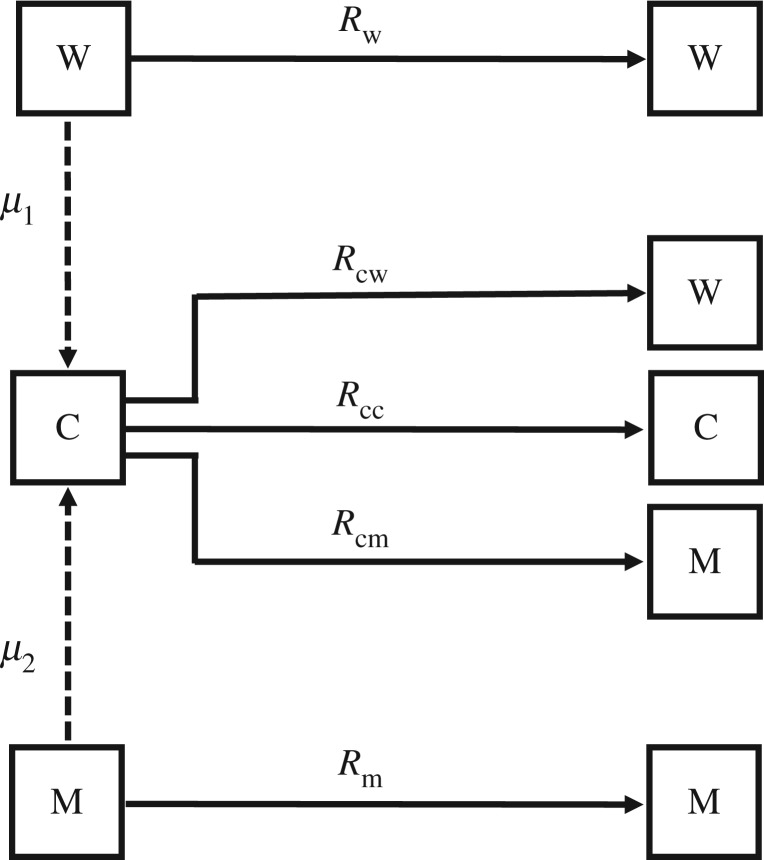
For each type of infection (wild-type, W; mutant, M; co-infection, C) the type(s) of progeny that can be generated are shown. Each arrow is labelled with the expected number of progeny of the type indicated.

We use a discrete-time multi-type branching process model (a type of Markov chain) to model the emergence of a mutant with *R*_0_ ≥ 1 from a wild-type ancestor with *R*_0_ ≤ 1. The model assumes three types of infections: wild-type, mutant and co-infection (figure 7), which have *R*_0_ values denoted $R_{w}$, $R_{m}$ and $R_{c}$, respectively (see table 2 for a list of all model parameters). We assume $R_{w}\leq 1$, $R_{m}\geq 1$ and $R_{c}=dR_{m}+(1-d)R_{w}$, where *d* is the dominance of the mutant.

The branching process (figure 7) begins with a single wild-type infection. The mutant may arise (with probability $\mu_{1}$) in the course of a wild-type infection; if this occurs, the infection is re-classified as a co-infection. If the mutant does not arise, the wild-type infection leaves only wild-type progeny; the number of progeny is Poisson distributed with the mean $R_{w}$. If the mutant does arise, the resulting co-infection can leave progeny of all three types: wild-type, mutant and co-infection.

The proportions of each type are determined by parameters *c* (the probability of co-transmission of wild-type and mutant) and *b* (the probability that, if only one type is transmitted, it is the mutant). The numbers of wild-type, mutant and co-infection progeny are Poisson distributed with means $R_{\mathrm{cw}},R_{\mathrm{cm}}$ and $R_{\mathrm{cc}}$, where

$$R_{\mathrm{cc}}=cR_{c},$$

$$R_{\mathrm{cm}}=(1-c)bR_{c}$$

$$\mathrm{and}\;\;\;\;R_{\mathrm{cw}}=(1-c)(1-b)R_{c}.\;\;$$

A mutant infection behaves similarly to a wild-type infection: the mutant can revert to the wild-type with probability $\mu_{2}$, in which case the infection is re-classified as a co-infection and can leave progeny of all three types, as described above. If reversion does not occur, the mutant leaves only mutant progeny; the number of progeny is Poisson distributed with the mean $R_{m}$.

We use probability generating functions to calculate the extinction probability for a branching process beginning with a single wild-type infection (refer to electronic supplementary material, text S1 for details). Here, we assume that *P*(emergence) = 1 – *P*(extinction). We use Latin hypercube sampling (R package *pse*) to examine the effect of dominance on the probability of emergence. This method divides each parameter range into *N* equal intervals (in this case, *N* = 500) and draws a random parameter value from each interval, resulting in *N* randomly generated sets of parameters which are used as inputs for the model. We then compute the partial rank correlation coefficient (PRCC) to describe the association between the randomly generated values of *d* (dominance) and the corresponding emergence probabilities, and use a total of 500 bootstrap replicates to estimate a 95% confidence interval for the PRCC (R package *sensitivity*). The advantage of Latin hypercube sampling is that, by varying all model parameters simultaneously, we ensure that the observed effect of dominance is not influenced by the choice of values for the other parameters in the model; the PRCC factors out the influence of the additional parameters on emergence probability, and has the additional advantage of being non-parametric (does not assume the correlation between two variables is linear).

For the remainder of the main text, we limit our analysis to mutants that are either recessive (d=0) or dominant (d=1); the electronic supplementary material contains a more complete analysis in which *d* is varied over the interval [−0.5,1.5] to explore the effects of underdominance, incomplete dominance and overdominance. We use the Latin hypercube sampling approach again for dominant and recessive mutants in order to assess the overall impact of each model parameter on emergence in each case. As above, these effects are quantified using PRCCs. We also vary each of the model parameters individually (while holding the other parameters constant) in order to explore in more detail how each parameter affects emergence and how these effects differ for dominant and recessive mutants.

## Supplementary Material

Fig. S1 - Dominance of hypothetical mutant phenotypes

## Supplementary Material

Fig. S2 - Within-host dynamics of mutants with increased replication rates

## Supplementary Material

Fig. S3 - Within-host dynamics of mutants with decreased replication rates

## Supplementary Material

Fig. S4 - Within-host dynamics of mutants with decreased virulence

## Supplementary Material

Fig. S5 - Effect of dominance on probability of emergence

## Supplementary Material

Fig. S6 - Probability of emergence vs. dominance for varying parameter values

## Supplementary Material

Fig. S7 - Probability of emergence vs. parameter values for varying dominance

## Supplementary Material

Text S1 - Branching process model

## Data accessibility

The source code for the models (including all results) is available through Open Science Framework at https://osf.io/2kzs3/.
