# Supplementary figures and images for: A general framework for modelling the impact of co-infections on pathogen evolution
Source: J R Soc Interface. 2019 Jun 26;16(155):20190165. doi: 10.1098/rsif.2019.0165 (PMC6597765; doi:10.1098/rsif.2019.0165)

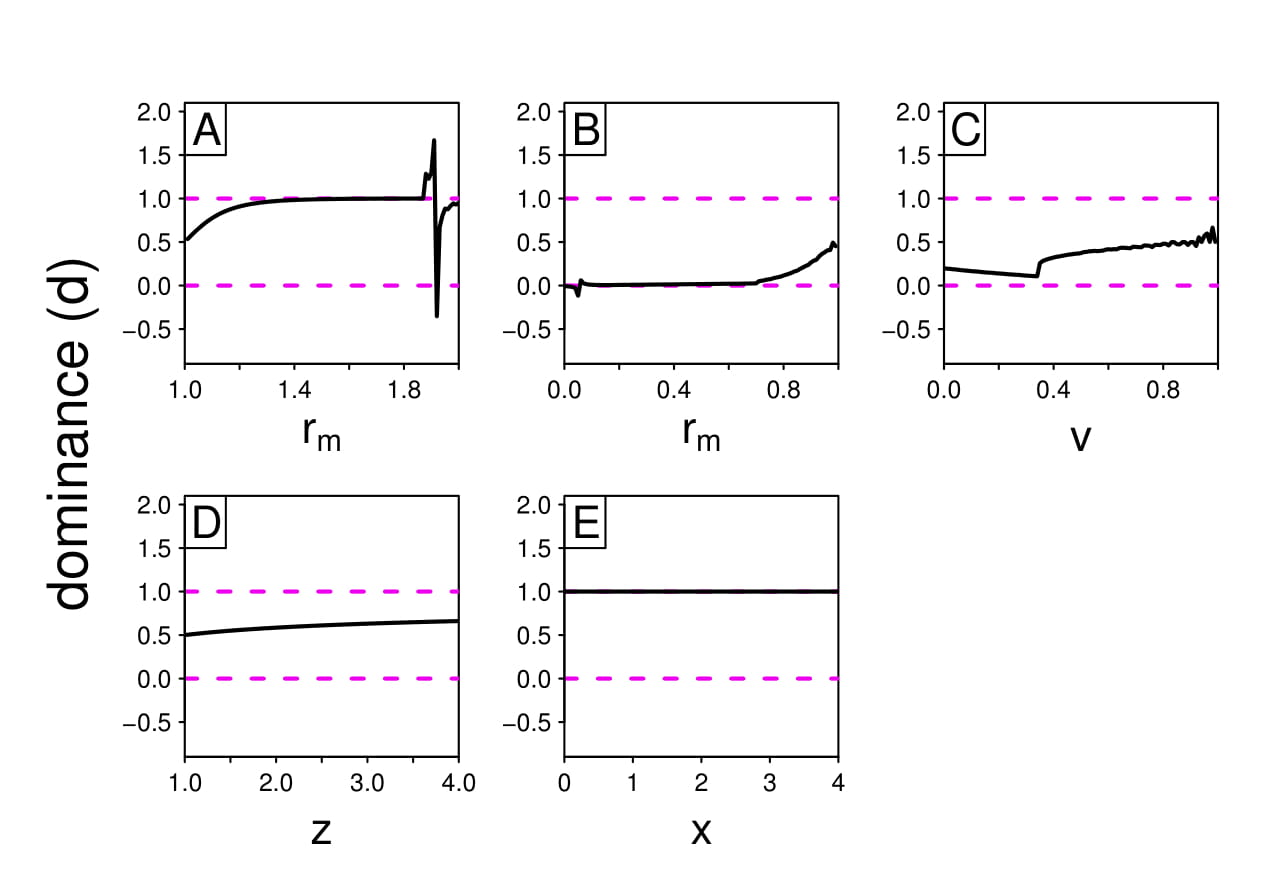

Supplement: Fig. S1 - Dominance of hypothetical mutant phenotypes [file rsif20190165supp1.jpg]

$\lambda$  (transmission) =  $\log_{10}\alpha$  (pathology)

mutant alone

coinfection

$r_m = 1.5$

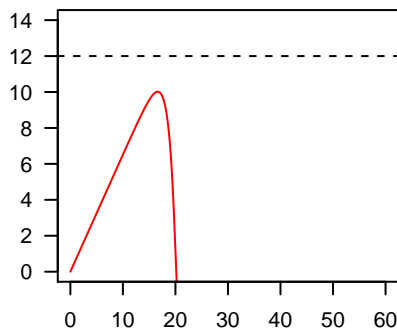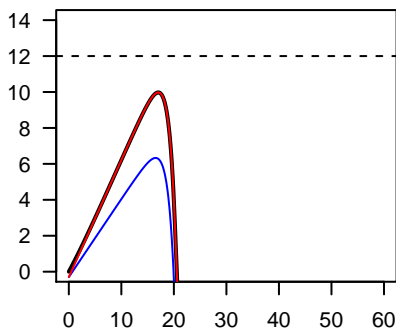

$r_m = 1.8$

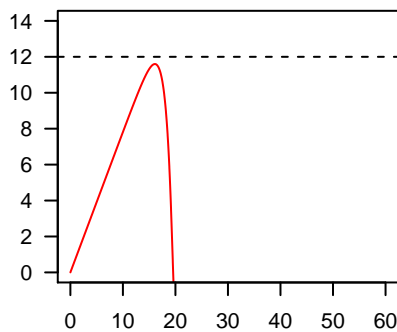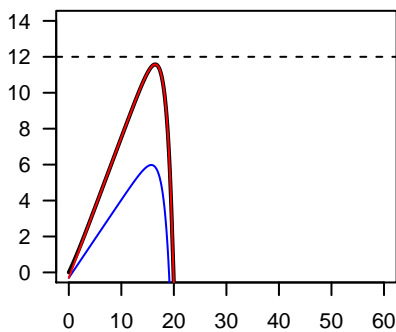

$r_m = 1.9$

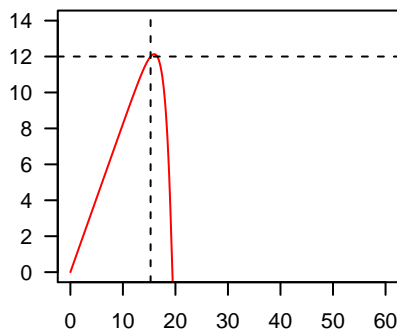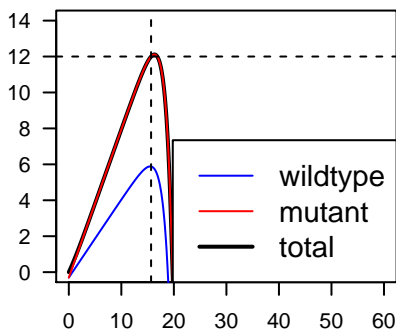

time (days)

Supplement: Fig. S2 - Within-host dynamics of mutants with increased replication rates [file rsif20190165supp2.pdf]

$\lambda$  (transmission) =  $\log_{10}\alpha$  (pathology)

mutant alone

coinfection

$r_m = 0.2$

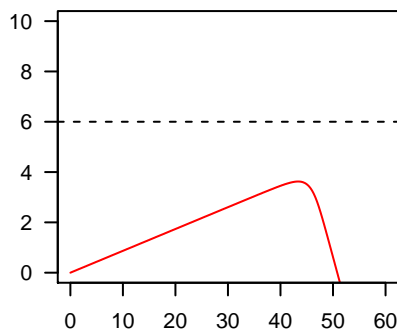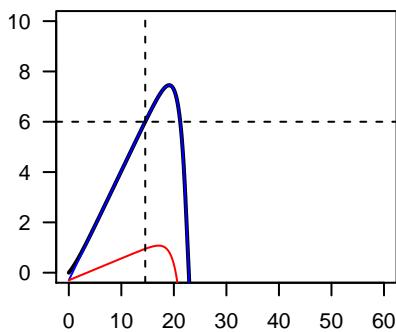

$r_m = 0.6$

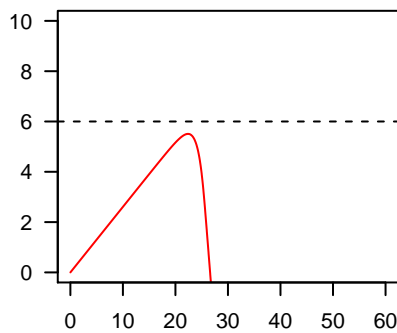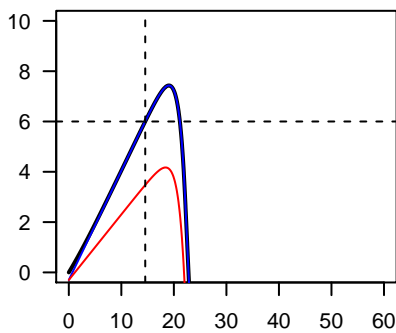

$r_m = 0.8$

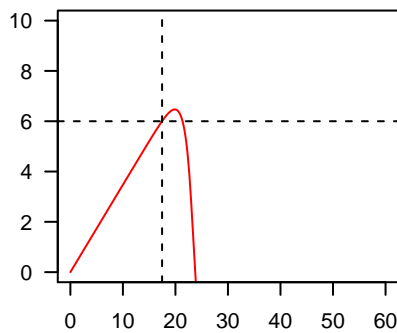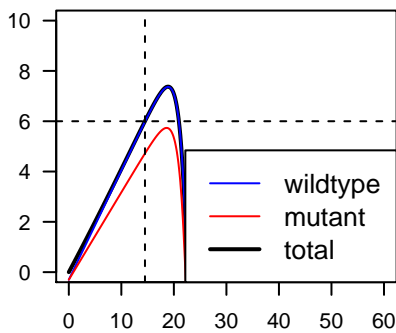

time (days)

Supplement: Fig. S3 - Within-host dynamics of mutants with decreased replication rates [file rsif20190165supp3.pdf]

$\log_{10}\alpha$  (pathology)

mutant alone

coinfection

$v = 0.2$

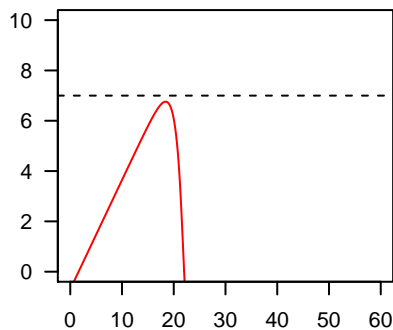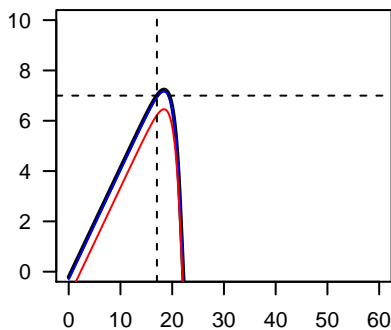

$v = 0.4$

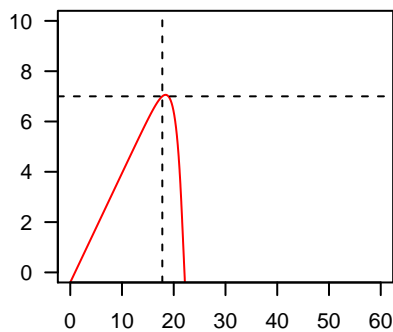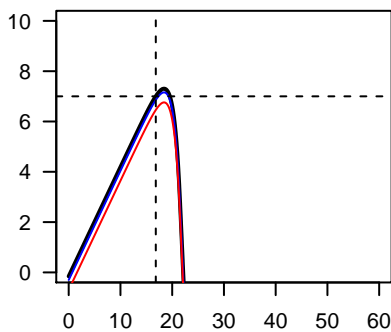

$v = 0.7$

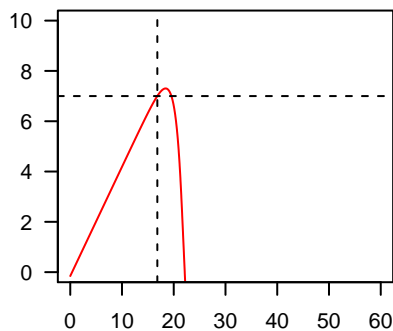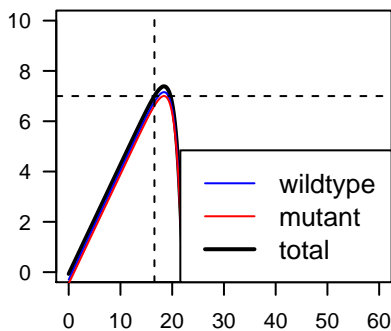

time (days)

Supplement: Fig. S4 - Within-host dynamics of mutants with decreased virulence [file rsif20190165supp4.pdf]

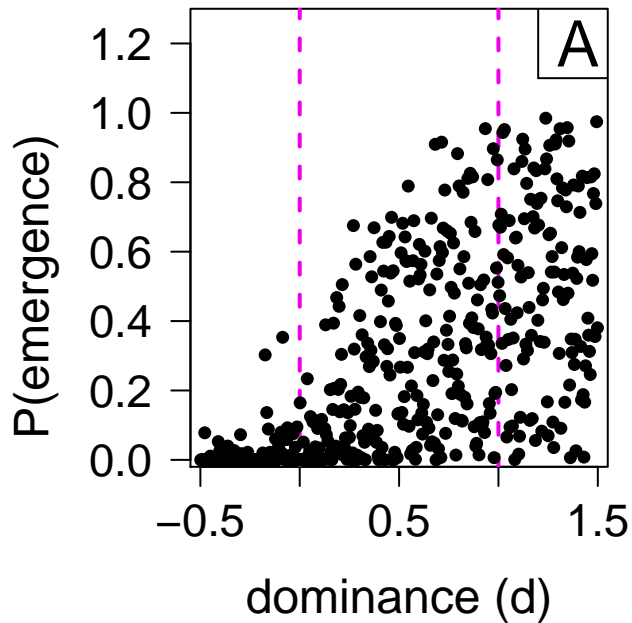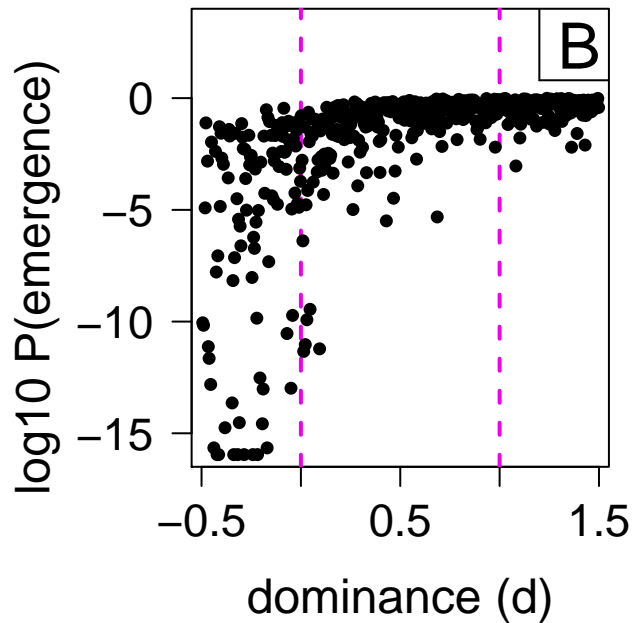

Supplement: Fig. S5 - Effect of dominance on probability of emergence [file rsif20190165supp5.pdf]

$\log_{10}$  probability of emergence

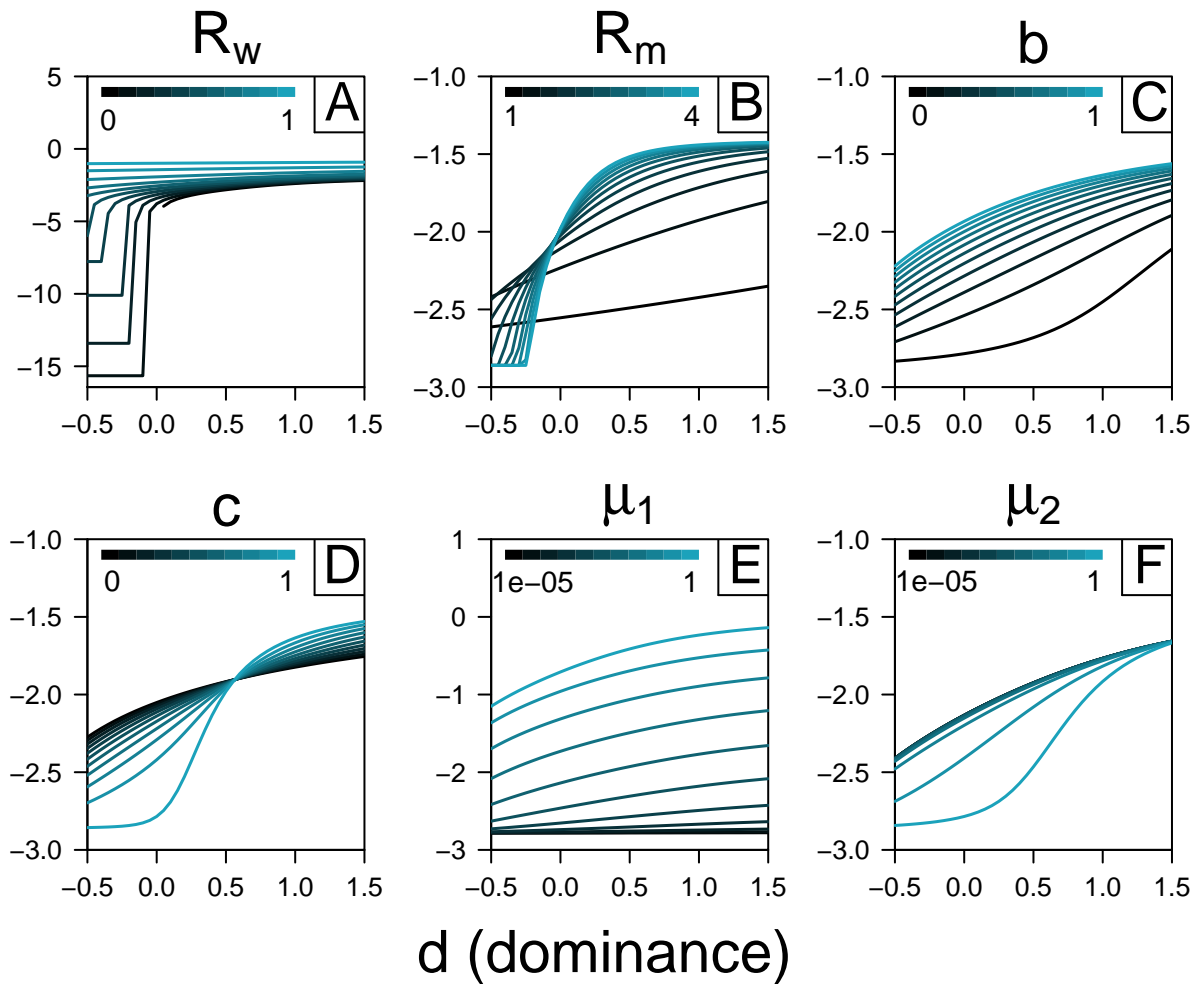

Supplement: Fig. S6 - Probability of emergence vs. dominance for varying parameter values [file rsif20190165supp6.pdf]

$\log_{10}$  probability of emergence

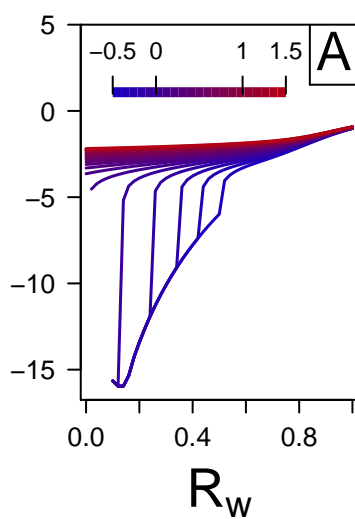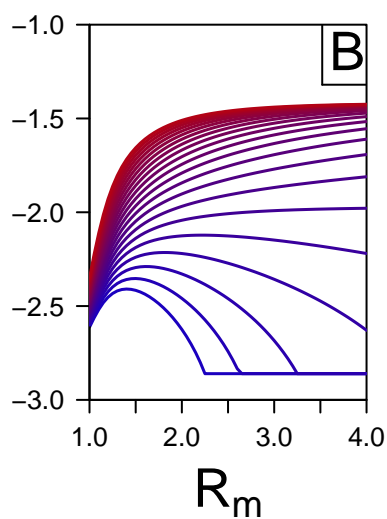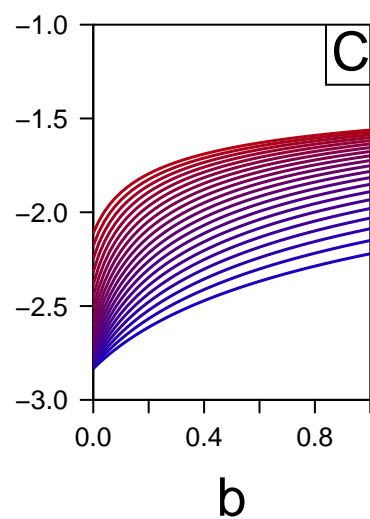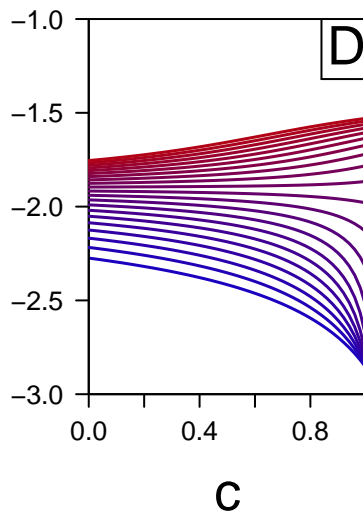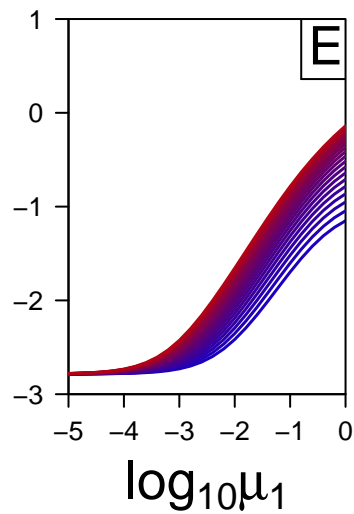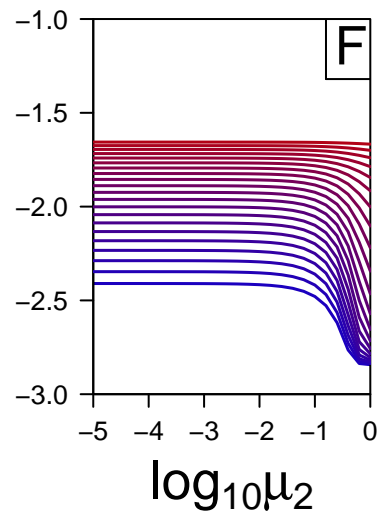

Supplement: Fig. S7 - Probability of emergence vs. parameter values for varying dominance [file rsif20190165supp7.pdf]
