# Supplementary material for: A general framework for modelling the impact of co-infections on pathogen evolution
Source: J R Soc Interface. 2019 Jun 26;16(155):20190165. doi: 10.1098/rsif.2019.0165 (PMC6597765; doi:10.1098/rsif.2019.0165)
Supplement: Text S1 - Branching process model [file rsif20190165supp8.pdf]

# Multi-type branching process model

*Mary Bushman*

*July 21, 2018*

Define a multitype branching process with $X(n) = (X_w(n), X_m(n), X_c(n))$ where $X_w(n)$, $X_m(n)$, and $X_c(n)$ are the number of wildtype infections, mutant infections, and coinfections (wildtype + mutant) at time step $n$.

Let $R_w$ and $R_m$ represent the $R_0$ values for wildtye and mutant infections, respectively. Let $R_c$ be the $R_0$ value for a coinfection (wildtype + mutant) , with $R_c = dR_m + (1-d)R_w$ where $d$ is the "dominance" of the mutant genotype.

Let $c$ be the probability that both wildtype and mutant are transmitted from a coinfection. In the event that only one strain is transmitted from a coinfection, let $b$ be the probability that it is the mutant.

Let $Y_{ij}$ be the number of progeny of type $j$ from an individual of type $i$. Then let

$Y_{ww} \sim \text{Poisson}(R_w)$
$Y_{mm} \sim \text{Poisson}(R_m)$
$Y_{cw} \sim \text{Poisson}((1-b)(1-c)R_c)$
$Y_{cm} \sim \text{Poisson}(b(1-c)R_c)$
$Y_{cc} \sim \text{Poisson}(cR_c)$

All other $Y_{ij}$ are assumed to be zero.

Define $R_{ij} = \mathrm{E}[Y_{ij}]$.

Let $\mu_1$ be the mutation probability (probability that a wildtype infection becomes a coinfection) and let $\mu_2$ be the reversion probability (probability that a mutant infection becomes a coinfection).

Let $p_w(k_w, k_m, k_c)$ be the probability that a wildtype infection gives rise to $k_w$ wildtype infections, $k_m$ mutant infections and $k_c$ coinfections. To find this probability it is necessary to condition on whether a mutation occurs in the wildtype infection, turning it into a coinfection. Thus,

$$p_w(k_w, k_m, k_c) = (1-\mu_1)Pr(k_w, k_m, k_c | \text{ no mutation}) + \mu_1 Pr(k_w, k_m, k_c | \text{ mutation})$$

.

For shorthand, define $z(x) = \begin{cases} 1 & \text{if } x = 0 \\ 0 & \text{otherwise} \end{cases}$

Thus, we have

$$p_w(k_w, k_m, k_c) = z(k_m)z(k_c)(1-\mu_1)\frac{R_{ww}^{k_w} e^{-R_{ww}}}{k_w!} + \mu_1 \frac{R_{cw}^{k_w} e^{-R_{cw}}}{k_w!} \frac{R_{cm}^{k_m} e^{-R_{cm}}}{k_m!} \frac{R_{cc}^{k_c} e^{-R_{cc}}}{k_c!}$$

Similarly,

$$p_m(k_w, k_m, k_c) = z(k_w)z(k_c)(1-\mu_2)\frac{R_{mm}^{k_m} e^{-R_{mm}}}{k_m!} + \mu_2 \frac{R_{cw}^{k_w} e^{-R_{cw}}}{k_w!} \frac{R_{cm}^{k_m} e^{-R_{cm}}}{k_m!} \frac{R_{cc}^{k_c} e^{-R_{cc}}}{k_c!}$$

$$p_c(k_w, k_m, k_c) = \frac{R_{cw}^{k_w} e^{-R_{cw}}}{k_w!} \frac{R_{cm}^{k_m} e^{-R_{cm}}}{k_m!} \frac{R_{cc}^{k_c} e^{-R_{cc}}}{k_c!}$$

We then use these probability distributions to define the probability generating functions:

$$f_w(t_w, t_m, t_c) = \sum_{k_w=0}^{\infty} \sum_{k_m=0}^{\infty} \sum_{k_c=0}^{\infty} p_w(k_w, k_m, k_c) t_w^{k_w} t_m^{k_m} t_c^{k_c}$$

$$f_m(t_w, t_m, t_c) = \sum_{k_w=0}^{\infty} \sum_{k_m=0}^{\infty} \sum_{k_c=0}^{\infty} p_m(k_w, k_m, k_c) t_w^{k_w} t_m^{k_m} t_c^{k_c}$$

$$f_c(t_w, t_m, t_c) = \sum_{k_w=0}^{\infty} \sum_{k_m=0}^{\infty} \sum_{k_c=0}^{\infty} p_c(k_w, k_m, k_c) t_w^{k_w} t_m^{k_m} t_c^{k_c}$$

Each generating function maps $[0,1]^3 \to [0,1]$.

Then denote $g(t_w, t_m, t_c) = (f_w(t_w, t_m, t_c), f_m(t_w, t_m, t_c), f_c(t_w, t_m, t_c))$.

Note that $g(t_w, t_m, t_c)$ maps $[0,1]^3 \to [0,1]^3$.

If we select some values $t_w, t_m, t_c \in [0,1)$ and call these $t_w(0), t_m(0), t_c(0)$, and define $(t_w(n), t_m(n), t_c(n)) = g(t_w(n-1), t_m(n-1), t_c(n-1))$ then

$$\lim_{n\to\infty} (t_w(n), t_m(n), t_c(n)) = (q_w, q_m, q_c)$$

is a fixed point for $g(t_w, t_m, t_c)$ on $[0,1]^3$. Furthermore, $q_i$ is the probability of eventual extinction for a multitype branching process beginning with a single infection of type $i$. For a multitype branching process beginning with $(x_w, x_m, x_c)$ wildtype, mutant and coinfections, the ultimate extinction probability is

$$q_w^{x_w} q_m^{x_m} q_c^{x_c}$$

Approximate calculations of $f_w(t_w, t_m, t_c)$, $f_m(t_w, t_m, t_c)$, and $f_c(t_w, t_m, t_c)$ are easy to implement in R, as is the algorithm to converge to the fixed point of the function $g(t_w, t_c, t_m)$. Thus, for any set of parameters $(R_w, R_m, d, c, b, \mu_1, \mu_2)$ we can easily calculate the ultimate extinction probability for starting conditions $(x_w, x_m, x_c)$. (Generally we will assume $x_w = 1, x_m = x_c = 0$, but the calculations are equally straightforward for other starting conditions.)
